# Healing effects of topically applied *Ocimum basilicum* L. on excisional wounds in mice

**DOI:** 10.1590/acb403825

**Published:** 2025-06-06

**Authors:** Karine Sthéfany Serpa Amaral Dias, Elisângela Elduina Ferreira, Renan de Araújo Costa, Letícia Marcelle Ferreira, Renan Diniz Ferreira, Milena Santos de Almeida, Laura Kaori Meneguessi Nakano, Karen Helaine Mendes Bertolin, Luciana Alves Rodrigues dos Santos Lima, Raquel Alves Costa, Flávia Carmo Horta Pinto

**Affiliations:** 1 Universidade Federal de São João del-Rei – Laboratory of Experimental Pathology – São João del-Rei (MG) – Brazil.; 2 Universidade Federal de São João del-Rei – Laboratory of Pathology Anatomy – São João del-Rei (MG) – Brazil.; 3 Universidade Federal de São João del-Rei – Laboratory of Phytochemistry – Divinópolis (MG) – Brazil.; 4 Universidade Federal de São João del-Rei – Laboratory of Biology of Repair and Nanomaterials – São João del-Rei (MG) – Brazil.

**Keywords:** Wound Healing, Skin, Anti-Inflammatory Agents, Ocimum basilicum

## Abstract

**Purpose:**

To evaluate the topical treatment with the ethanolic extract of *Ocimum basilicum* L. (OBEE) on excisional wounds in mice.

**Methods:**

The plant material was identified and collected, and the ethanolic extract was obtained from the aerial parts of *O. basilicum*. The OBEE was resuspended in saline at concentrations of 38 and 100 mg/kg for topical application on the left and right lesions, respectively. Mice were anesthetized, excisional wounds were made on the dorsal region and divided into four groups (n = 6) based on treatment duration: one, three, five, and 21 days. The control group received saline. After the treatments, the animals were euthanized, and the wounds were collected for histopathological analysis the inflammatory infiltrate, mast cell count, and deposition of newly synthesized collagen matrix.

**Results:**

The daily topical application of OBEE at concentrations of 38 and 100 mg/kg reduced the inflammatory response, evidenced by decreased leukocyte infiltration in the damaged tissue, and improved the deposition of newly synthesized matrix, with thicker, more intertwined collagen fibers resembling intact skin. These effects may be attributed to the phenolic compounds, tannins, flavonoids, and essential oils present in basil.

**Conclusion:**

OBEE shows promise as a potential healing agent in excisional wounds.

## Introduction

Multicellular organisms have various molecular mechanisms to restore tissue architecture, ensuring that, in the event of damage, a new matrix is rapidly deposited to prevent the entry of microorganisms^1^. Nevertheless, therapies that accelerate or improve both the aesthetic and functional aspects of skin wound repair are still being sought^2^. The healing process in the skin and other organs involves three distinct but overlapping phases: an initial inflammatory phase, a proliferative phase, and a remodeling or reorganization phase of the newly synthesized matrix^3^.

Resolution of inflammation during wound healing has been reported in several studies as a crucial factor in preventing complications at the end of the healing process. The formation of hypertrophic and keloid scars has been also correlated with dysfunctional inflammatory processes^1^^,^ 4. Reducing oxidative stress and inflammation in the wound environment using various natural substances has been studied and identified as a means to improve healing^5^^–^ 7.

*Ocimum basilicum*, commonly known as basil, belongs to the family Lamiaceae and is widely used as a condiment with global distribution. Its nutraceutical potential is studied in various pathologies^8^^,^ 9. In popular medicine, it is used to treat renal problems, colds, menstrual irregularities, and inflammatory conditions such as arthritis and asthma^10^. The pharmacological value of basil includes antimicrobial^11^^,^ 12, antioxidant^13^^,^ 14, anti-inflammatory^15^^,^ 16, and antitumor^17^ effects. These effects are associated with the presence of phenolic compounds, tannins, flavonoids, and essential oils^13^.

In wound healing, *O. basilicum* has shown satisfactory effects and is commonly used in some regions. The species’ immunomodulatory potential has been linked to its healing activity for conditions like asthma and possibly its promising effects in wound repair^2^^,^ 18. Additionally, its antioxidant capacity may also contribute to improved healing^6^. Observed activities of this species in the healing process include accelerating wound closure, increasing contraction and re-epithelialization, stimulating fibroblast proliferation, and improving collagen remodeling^2^^,^ 19 ^,^ 20.

In this context, the present study aimed to evaluate whether the topical application of the ethanolic extract of *O. basilicum* affects the healing of excisional wounds in mice during different phases of the repair process.

## Methods

### Preparation of the ethanolic extract

The extract was obtained from the aerial parts of *O. basilicum*, which were collected in Carmópolis de Minas, Minas Gerais, Brazil. A specimen was deposited at the Herbarium of the Institute of Biological Sciences at the Universidade Federal de Minas Gerais, under registration No. BHCB 147240, and was identified by Alexandre Salino.

Basically, 234.08 grams of fresh plant material were ground and macerated in analytical-grade ethanol for 10 days. Afterwards, the material was filtered and dried using a rotary evaporator, resulting in 10.299 grams of ethanolic extract. This extract has been used in other studies and was previously characterized^21^^–^ 23. The solutions used for treating the animals were prepared by resuspending the extract in saline solution (0.9% NaCl) at concentrations of 38 and 100 mg/kg.

### Animals

Forty-eight male 8-week-old Swiss mice, each weighing approximately 40 g, were obtained from the Laboratory Animal Breeding Center of the Universidade Federal de São João del-Rei (UFSJ). During the experiments, the animals were housed in cages with six mice per cage under standard conditions of temperature (20 to 24°C) and a light/dark cycle, with solid food and water provided *ad libitum*. All procedures performed during the experiments were approved by the UFSJ Animal Ethics Committee (CEUA-UFSJ), under protocol No. 026/2019.

### Experimental groups and surgical procedure

The animals were randomly divided into two groups of 24 each: the control group, which received saline, and the treated group, which received the ethanolic extract of O. basilicum (OBEE). Each group was further subdivided into four subgroups based on the duration of treatment: one, three, five, and 21 days.

Two excisional wounds, each measuring 6.5 mm in diameter, were created on the dorsal region of the animals, which were previously anesthetized with xylazine hydrochloride (16.5 mg/kg) (Injectable Anasedan, Ceva), ketamine hydrochloride (97 mg/kg) (Injectable Dopalen, Ceva), and physiological saline, in a 1:1:2 ratio, respectively, following routine trichotomy^24^. The control group received daily topical administration of physiological saline (0.9% NaCl) on their lesions, applied three times daily. In the groups treated with the OBEE, the animals received 40 μL of the OBEE solution at the concentration of 38 mg/kg on the left-side wound and 105 μL at the concentration of 100 mg/kg on the right-side wound (standardized for all animals), with both treatments applied three times a day according to the corresponding treatment and euthanasia times: one, three, five, and 21 days.

### Macroscopic analysis

For the macroscopic evaluation of the excisional wounds, a digital camera mounted on a tripod was used to photograph the lesions of each animal on two occasions: on the day the excisional wounds were created, and on the day of euthanasia. Subsequently, all images were imported into the ImageJ image analysis software, version 1.44 (Research Services Branch, U.S. National Institutes of Health, Bethesda, MD, United States of America). The contours were manually traced to calculate the wound size in mm^2^ for all animals in each group at different time points (one, three, five, and 21 days) in order to assess closure and re-epithelialization. Wound sizes were then compared between groups.

### Histopathological analysis

After euthanasia, fragments containing the surgical wounds in their entirety and depth were removed for histopathological analysis. They were fixed in 10% buffered formalin, dehydrated in ethanol (70 to 100%), cleared in xylene, and embedded in paraffin. Sections measuring 4 µm thick were cut and stained with hematoxylin and eosin (HE), toluidine blue (TB), and Gomori’s trichrome (GT). Slides were prepared from each wound and evaluated for healing area, re-epithelialization, presence of inflammatory infiltrate, mast cells, and collagen deposition.

Quantitative analysis of leukocytes and mast cells (identified by their characteristic morphology) was performed after one, three, and five days of treatment using the sections stained with HE and TB, respectively. Images were generated with a light microscope (Motic) connected to a digital camera (Moticam 580 5.0 MP) linked to an on-board computer scanner. A total of 10 fields per case/slide was analyzed at 400x magnification in the wound healing area. The results were expressed per group as the number of cells per μm^2^. 

A qualitative analysis of the remodeling phase was conducted to assess whether treatment with OBEE affected collagen deposition after 21 days of treatment. The histological sections stained with HE and GT were analyzed across the entire healing area following criteria for the presence of inflammation, epithelial characterization/re-epithelialization, presence of nerve bundles, cells and vascularization, scar size, and collagen fiber arrangement. The images were generated with a light microscope (Motic) connected to a digital camera (Moticam 580 5.0 MP) linked to an on-board computer scanner.

### Statistical analyses

All data were expressed as mean and standard error of the mean and analyzed using GraphPad Prism 5 (GraphPad Software, CA, United States of America). One-way analysis of variance, followed by the Newman-Keuls post-hoc test, was performed to compare the three groups (Saline, OBEE at 38 mg/kg, and OBEE at 100 mg/kg). Results were considered significant when *p* < 0.05.

## Results

In order to relate the effects of *O. basilicum* and its actions in the different phases of wound healing (inflammation, proliferation, and remodeling), we applied topical treatments with OBEE at concentrations of 38 and 100 mg/kg to excisional wounds in mice. The wounds were evaluated both macroscopically and histopathologically at various time points (one, three, five, and 21 days) to assess the effects of basil on the healing process.

**Figure 1:**
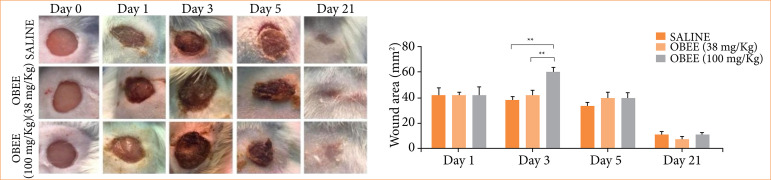
Effects of the ethanolic extract of Ocimum basilicum (OBEE) at concentrations of 38 and 100 mg/kg on the area of excisional lesions. **(a)** Macroscopic analysis of the wounds after one, three, five, and 21 days of treatment (n = 6). **(b)** Graph displaying the wound area in mm^2^. Statistical analysis revealed an increase in wound area in the group treated with *Ocimum basilicum* ethanolic extract (OBEE) 100 mg/kg after three days.

Figure 1 provides a comparative view of the wound areas at different time points following topical treatment. After three days, the wounds in the treated groups were relatively larger compared to the control group. However, subsequent wound closure over time showed no significant differences between groups.

**p < 0.01: Newman-Keuls test. Source: Elaborated by the authors.

**Figure 2:**
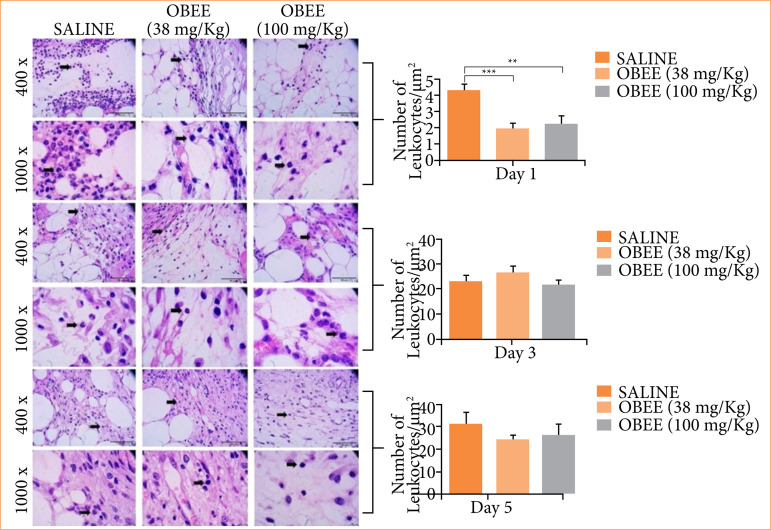
Effects of ethanol extract of *Ocimum basilicum* (OBEE) at concentrations of 38 and 100 mg/kg on the number of leukocytes after one, three, and five days of treatment (n = 6). **(a)** Histological photomicrographs of the different experimental groups during the analysis. Sections of skin were stained with hematoxylin and eosin. The arrows indicate leukocytes. The original magnification was 400X and 1,000X. **(b)** Quantitative analysis of inflammatory infiltrate showing a significant reduction in the OBEE groups compared to the control group after one day of treatment (*p* < 0.01: Newman-Keuls).

Source: Elaborated by the authors.

The wound healing process involves a series of histopathological changes over time. Thus, evaluations of leukocyte and mast cell infiltration during the inflammatory phase, as well as the deposition of newly synthesized matrix during the remodeling phase, were carried out. Figure 2 illustrates the assessment of inflammatory infiltrate in the wound bed after one, three, and five days of topical treatment with OBEE. A reduction in infiltrate was observed in the lesion area after one day of treatment compared to the control group, with the lowest content noted in the group treated with 38 mg/kg. At the other evaluated time points, the local inflammatory profile showed no statistically significant differences compared to the control group. However, a trend towards reduced inflammatory infiltrate in the tissue was observed.

**Figure 3:**
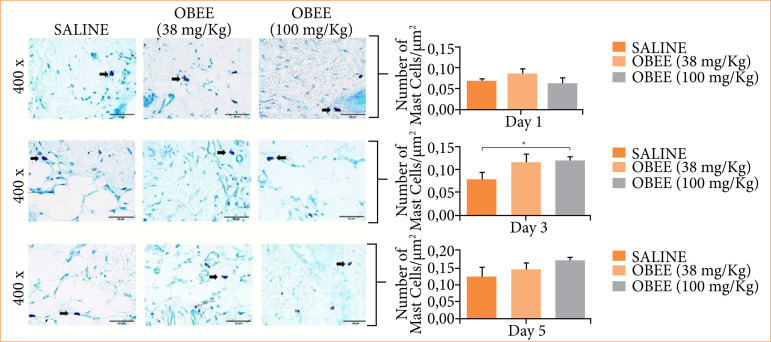
Effects off ethanol extract of *Ocimum basilicum* (OBEE) at concentrations of 38 and 100 mg/kg on the number of mast cells after one, tree and five days of treatment (n = 6). **(a)** Histological photomicrographs of the different experimental groups during the analysis. Sections of skin were stained with toluidine blue. The arrows indicate mast cells. The original magnification was 400X, and the bars represent 20 μm. **(b)** Statistical analysis revealed significant differences in the number of mast cells in the group treated with OBEE 100 mg/kg after three days.

Mast cell analysis was conducted using the histological sections stained with TB. These cells, identified by their dark blue staining, were found in greater numbers in the marginal areas of the lesion and in smaller quantities in the wound bed (Fig. 3a). The quantitative analysis of mast cells after one, three, and five days of treatment revealed no statistically significant differences compared to the control group (Fig. 3b).

*p < 0.05: Newman-Keuls test. Source: Elaborated by the authors.

**Figure 4:**
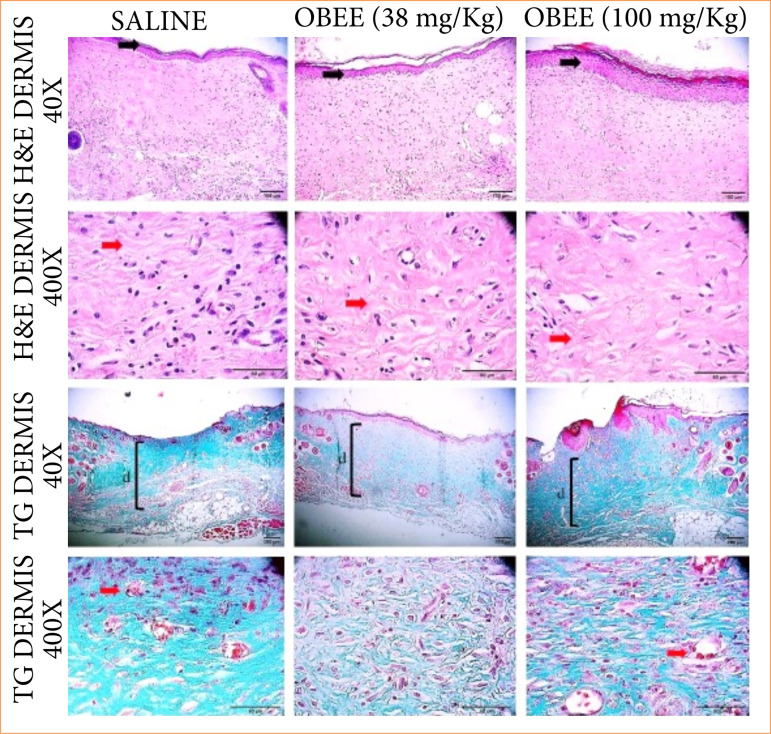
Effects of the ethanol extract of *Ocimum basilicum* (OBEE) at concentrations of 38 and 100 mg/kg on the new collagen matrix (n = 6). **(a)** Representative photomicrographs of the excisional wounds stained with hematoxylin and eosin, at 40X and 400X magnification for 21 days. The black arrows indicate complete re-epithelialization at the wound site for all groups; the red arrows indicate collagen fibers. **(b)** Histopathological aspects of the matrix during the analyzed periods. Sections of skin were stained with Gomori’s trichrome. The original magnification was 40X and 400X, and the bars represent 20 μm. The black bar indicates the location of the newly synthesized matrix; the red arrows indicate the presence of vascularization.

Source: Elaborated by the authors. Elaborated by the authors.

Figure 4 shows the histopathological analysis of the excisional wound areas after 21 days of treatment, aimed at qualitatively evaluating the remodeling phase and determining whether the treatment with OBEE could influence collagen deposition. Re-epithelialization was complete in all groups. The treated groups displayed newly formed dermis in the wound area with fewer cells and a higher number of blood vessels compared to the control group (Fig. 4a). In the histological sections stained with GT, the saline group exhibited a more aligned and less interlaced arrangement of collagen fibers. Conversely, the deposition of newly synthesized matrix (Fig. 4b) in the animals treated topically with OBEE exhibited a better arrangement of collagen fibers–thicker, more interlaced, and irregularly directed–resembling the appearance of intact skin (normal skin).

## Discussion

In response to an injury, a series of events occur in the skin to promote repair. Wound healing ensures the production of a new matrix and structural recovery, although tissue functionality may not be always fully restored. Sebaceous glands and hair follicles do not regenerate, and the newly deposited matrix does not exhibit the same characteristics as intact skin, presenting with less interlaced bundles and less dense collagen fibers^3^. The response to injury is rapid, although factors such as uncontrolled inflammation, oxidative stress, and microbial infections can negatively impact this process^1^.

In this study, we assessed the effects of topical application of *O. basilicum* L. on excisional wound healing in mice, aiming to compare these effects and relate them to the anti-inflammatory, antioxidant, and antimicrobial potentials already described in the literature for this species. The reduction of initial inflammatory response and the improved deposition of collagen fibers in the newly synthesized matrix after the treatment are among our main findings.

The reduction of inflammation during wound healing has been associated with better outcomes in repair^7^^,^ 25 ^–^ 28. The injured microenvironment, in response to damage, is initially rich in pro-inflammatory mediators such as interleukin (IL)-1, IL-6, and tumor necrosis factor (TNF)-α, which stimulate leukocytes to remove potential pathogens and cellular debris. Prolonged and excessive inflammatory reactions are commonly associated with tissue damage and delayed healing^29^.

The reduction in leukocyte infiltrate reported herein by *O. basilicum* is consistent with our previous studies6 and reinforces the potential of this species in this context. In line with our results, a study conducted by Zangeneh et al.^2^ demonstrated a decrease in leukocytes in animals with lesions treated with a 3% aqueous extract ointment of *O. basilicum* at 10, 20, and 30 days post-injury. Additionally, Rakha et al.^30^ found that treatment with the ethanolic fraction of *O. basilicum* seeds at 400 mg/kg had an anti-inflammatory effect in a carrageenan-induced paw edema model.

Mast cells are involved in different phases of the healing process. They modulate inflammation, stimulate new vessel formation, and contribute to collagen matrix deposition and remodeling^31^. Although we did not find statistically significant differences in the number of these cells, a slight increase was noted in lesions treated with *O. basilicum*, which may have partly contributed to the observed results during the healing process in this study, including the modulation of the initial inflammatory response.

Among the compounds identified in the *O. basilicum* extract, there are steroids, triterpenes, flavonoids, alkaloids, coumarins, phenolic compounds, and volatiles^21^^–^ 23. These compounds have been reported to be responsible for the effects of *O. basilicum* in various dermal pathologies, including wound healing^20^. The species’ antioxidant and antimicrobial properties seem to enhance its efficacy in wound healing. The antioxidant action reduces oxidative stress in the wound, preventing cellular damage and promoting tissue regeneration. Additionally, studies observing the anti-inflammatory effects have also reported overall improvements in subsequent repair events^2^^,^ 19. These bioactive compounds, especially flavonoids, can act as anti-inflammatory agents by modulating the immune response and reducing the release of pro-inflammatory mediators such as TNF-α and IL-6^32^.

The assessment of collagen deposition after 21 days of treatment with *O. basilicum* in this study revealed that the architecture of the newly deposited matrix resembled normal dermis more closely, with a higher density of fibers in the restored area. Although the remodeling phase can last from weeks to months, this phase appeared advanced and had a better aspect in the type of lesion studied herein. The presented results confirm our hypothesis that the ethanolic extract of *O. basilicum* can influence different stages of the wound healing process, consistent with findings reported in the literature.

The effects of *O. basilicum* on wound healing demonstrated in this study may be related to the synergistic action of its phytoconstituents. This species is widely used around the world, both as a condiment and for its phytotherapeutic properties. Scientific studies emphasize the importance of recognizing its potential and traditional knowledge regarding its effects and encourage the development of formulations for wound healing^19^^,^ 33.

## Conclusion

The obtained results indicate that the daily topical administration of the OBEE effectively reduced the initial inflammatory response and improved collagen deposition during the healing process of excisional wounds in mice. However, in order to fully understand its mechanisms, further studies are needed to investigate the cellular signaling pathways involved in modulating this response, as well as preclinical and clinical studies to evaluate the efficacy and safety of *O. basilicum* in both animal and human models.

## Data Availability

All data sets were generated or analyzed in the current study.

## References

[1] Eming SA, Wynn TA, Martin P (2017). Inflammation and metabolism in tissue repair and regeneration. Science.

[2] Zangeneh MM, Zangeneh A, Seydi N, Moradi R (2019). Evaluation of cutaneous wound healing activity of *Ocimum basilicum* aqueous extract ointment in rats. Comp Clin Pathol.

[3] Gurtner GC, Werner S, Barrandon Y, Longaker MT (2008). Wound repair and regeneration. Nature.

[4] Larouche J, Sheoran S, Maruyama K, Martino MM (2018). Immune regulation of skin wound healing: mechanisms and novel therapeutic targets. Adv Wound Care (New Rochelle).

[5] Melguizo-Rodríguez L, de Luna-Bertos E, Ramos-Torrecillas J, Illescas-Montesa R, Costela-Ruiz VJ, García-Martínez O (2021). Potential effects of phenolic compounds that can be found in olive oil on wound healing. Foods.

[6] Schmitt C, Jacques MC, Andrighetti TT, Antunes AS, Barros CF, Freitas CMD, Bunhak EJ, Lima IA (2022). Hydroxypropyl methylcellulose-based films incorporated with *Ocimum basilicum* L. extract for skin wound healing. Concil.

[7] Costa R, Dias K, Rabelo G, Ferreira E, Ferreira L, Carvalho B, Silva I, Costa R, Lima L, Pinto F (2023). Anti-inflammatory effect of ethanolic extract of *Ocimum basilicum* L. in the healing process of incisional wounds in mice. Indian J Expl Biol.

[8] Sestili P, Ismail T, Calcabrini C, Guescini M, Catanzaro E, Turrini E, Layla A, Akhtar S, Fimognari C (2018). The potential effects of *Ocimum basilicum* on health: a review of pharmacological and toxicological studies. Expert Opin Drug Metab Toxicol.

[9] Dhama K, Sharun K, Gugjoo MB, Tiwari R, Alagawany M, Iqbal Yatoo M, Chaiumpa W, Michalak I, Elnesr SS, Farag MR (2023). A comprehensive review on chemical profile and pharmacological activities of Ocimum basilicum. Food Rev Int.

[10] Shahrajabian MH, Sun W, Cheng Q (2020). Chemical components and pharmacological benefits of Basil (*Ocimum basilicum*): A review. Int J Food Prop.

[11] Silva VA, Sousa JP, Pessôa HLF, Freitas AFR, Coutinho HDM, Alves LBN, Lima EO (2016). *Ocimum basilicum*: Antibacterial activity and association study with antibiotics against bacteria of clinical importance. Pharm Biol.

[12] Cai M, Wang Y, Wang R, Li M, Zhang W, Yu J, Hua R (2022). Antibacterial and antibiofilm activities of chitosan nanoparticles loaded with *Ocimum basilicum* L. essential oil. Int J Biol Macromol.

[13] Do TH, Truong HB, Nguyen HC (2020). Optimization of extraction of phenolic compounds from *Ocimum basilicum* leaves and evaluation of their antioxidant activity. Pharm Chem J.

[14] Qamar F, Sana A, Naveed S, Faizi S (2023). Phytochemical characterization, antioxidant activity and antihypertensive evaluation of *Ocimum basilicum* L. in l-NAME induced hypertensive rats and its correlation analysis. Heliyon.

[15] Rodrigues LB, Martins AOBPB, Ribeiro-Filho J, Cesário FRAS, Castro FF, Albuquerque TR, Fernandes MNM, Silva BAF, Quintans LJ, Araújo AAS, Menezes PDP, Nunes PS, Matos IG, Coutinho HDM, Goncalves Wanderley A, Menezes IRA (2017). Anti-inflammatory activity of the essential oil obtained from *Ocimum basilicum* complexed with β-cyclodextrin (β-CD) in mice. Food Chem Toxicol.

[16] Beltrán-Noboa A, Proaño-Ojeda J, Guevara M, Gallo B, Berrueta LA, Giampieri F, Perez-Castillo Y, Battino M, Álvarez-Suarez JM, Tejera E (2022). Metabolomic profile and computational analysis for the identification of the potential anti-inflammatory mechanisms of action of the traditional medicinal plants *Ocimum basilicum* and *Ocimum tenuiflorum*. Food Chem Toxicol.

[17] Anusmitha KM, Aruna M, Job JT, Narayanankutty A, Benil PB, Rajagopal R, Alfarhan A, Barcelo D (2022). Phytochemical analysis, antioxidant, anti-inflammatory, anti-genotoxic, and anticancer activities of different Ocimum plant extracts prepared by ultrasound-assisted method. Physiol Mol Plant Pathol.

[18] Eftekhar N, Moghimi A, Mohammadian Roshan N, Saadat S, Boskabady MH (2019). Immunomodulatory and anti-inflammatory effects of hydro-ethanolic extract of *Ocimum basilicum* leaves and its effect on lung pathological changes in an ovalbumin-induced rat model of asthma. BMC Complement Altern Med.

[19] Khan BA, Ullah S, Khan MK, Alshahrani SM, Braga VA (2020). Formulation and evaluation of *Ocimum basilicum*-based emulgel for wound healing using animal model. Saudi Pharm J.

[20] Antonescu IA, Antonescu A, Miere F, Fritea L, Teușdea AC, Vicaș L, Brihan I, Domuta M, Zdrinca M, Zdrinca M, Cavalu S (2021). Evaluation of wound healing potential of novel hydrogel based on *Ocimum basilicum* and *Trifolium pratense* extracts. Processes.

[21] Araújo SG, Lima WG, Pinto MEA, Morais MÍ, de Sá NP, Johann S, Santos Lima LAR (2019). Pharmacological prospection in-vitro of Lamiaceae species against human pathogenic fungi associated to invasive infections. Biocatal Agric Biotechnol.

[22] Araújo SG, Amado PA, Pinto MEA, Castro AHF, Lima LDS (2019). Total phenol and antioxidant potential of five species of Lamiaceae family. Tchê Química.

[23] Araújo SG, Alves LF, Pinto MEA, Oliveira GT, Siqueira EP, Ribeiro RIMA, Ferreira JMS, Lima LARS (2014). Volatile compounds of Lamiaceae exhibit a synergistic antibacterial activity with streptomycin. Braz J Microbiol.

[24] Moreira CF, Cassini-Vieira P, da Silva MF, Barcelos LS (2015). Skin wound healing model: excisional wounding and assessment of lesion area. Bio-protocol.

[25] Estevão LRM, Cassini-Vieira P, Leite AGB, Bulhões AAVC, Barcelos LS, Evêncio-Neto J (2019). Morphological evaluation of wound healing events in the excisional wound healing model in rats. Bio-protocol.

[26] Interdonato L, Marino Y, Franco GA, Arangia A, D’Amico R, Siracusa R, Cordaro M, Impellizzeri D, Fusco R, Cuzzocrea S, Paola RD (2023). Açai berry administration promotes wound healing through Wnt/β-catenin pathway. Int J Mol Sci.

[27] Short WD, Rae M, Lu T, Padon B, Prajapati TJ, Faruk F, Olutoye OO 2nd, Yu L, Bollyky P, Keswani SG, Balaji S (2023). Endogenous interleukin-10 contributes to wound healing and regulates tissue repair. J Surg Res.

[28] Xu X, Zeng Y, Chen Z, Yu Y, Wang H, Lu X, Zhao J, Wang S (2023). Chitosan-based multifunctional hydrogel for sequential wound inflammation elimination, infection inhibition, and wound healing. Int J Biol Macromol.

[29] Rodrigues M, Kosaric N, Bonham CA, Gurtner GC (2019). Wound healing: A cellular perspective. Physiol Rev.

[30] Rakha P, Sharma S, Parle M (2010). Anti-inflamatory potencial of the seeds of *Ocimum basilicum* Linn. in rats. Asian J Biol Sci.

[31] da Silva EZ, Jamur MC, Oliver C (2014). Mast cell function: a new vision of an old cell. J Histochem Cytochem.

[32] Prokopidis K, Mazidi M, Sankaranarayanan R, Tajik B, McArdle A, Isanejad M (2023). Effects of whey and soy protein supplementation on inflammatory cytokines in older adults: a systematic review and meta-analysis. Br J Nutr.

[33] Azizah NS, Irawan B, Kusmoro J, Safriansyah W, Farabi K, Oktavia D, Doni F, Miranti M (2023). Sweet basil (*Ocimum basilicum* L.): a review of its botany, phytochemistry, pharmacological activities, and biotechnological development. Plants (Basel).

